# Perceptions of mental health care consumers regarding their conditions

**DOI:** 10.4102/sajpsychiatry.v24i0.1317

**Published:** 2018-10-25

**Authors:** Meent F. Potgieter, Francois C. van Rooyen

**Affiliations:** 1 Department of Psychiatry, University of the Free State, South Africa; 2 Faculty of Health Sciences, Department of Biostatistics, University of the Free State, South Africa

## Abstract

**Background:**

Distress is experienced, understood, expressed and communicated differently across various cultures. Individuals assign meaning to their mental health problems from their own personal, social and cultural context. Mental healthcare consumers (MHCCs) often attribute their symptoms to a cultural or spiritual cause, and as a result, tend to visit spiritual or traditional healers before attending psychiatric units.

**Aim:**

The aim of the study was to determine patients’ perspectives on mental health – what they perceived to be the cause of mental problems and what they believed the most appropriate treatment options would be. The role of culture, religion and spirituality in patients’ understanding of and coping with mental problems was explored, as well as their attitudes toward their treatment and the perceived effectiveness and appropriateness of the different treatment modalities.

**Methods:**

A quantitative survey was done using a questionnaire designed to explore and analyse patients’ cultural and religious beliefs about mental illness and the actions they take in their search for recovery from mental disturbances. The questionnaire was compiled by incorporating demographic information, together with certain items of the World Health Organisation Quality of Life Spiritual, Religious and Personal Beliefs questionnaire, the Cultural Formulation Interview, the Rating of Medication Influences Scale, and the South African Traditional Beliefs Scale.

**Results:**

Ninety-four patients (58.5% male) were included in the study, with a mean age of 36 years. The majority of the participants (75.5%) were black people; most were Christian (79.8%), followed by African traditionalists (17.0%), and 41.4% indicated that they were actively involved in their religious communities. Although most (72.0%) believed that faith in God and help from religious leaders (34.4%) could contribute to their mental wellbeing, 29.0% reckoned that keeping their ancestors happy would protect them from sickness and bad luck. Approximately one quarter (22.3%) believed that traditional medicine could be the only cure for mental illness related to bewitchment, and 29.0% believed that Western medicine could worsen such problems. Roughly a third of participants (30.9%) were of the opinion that Western medicine cannot cure mental illness caused by angry ancestors.

**Conclusion:**

The impact cultural and religious belief systems have on MHCCs’ perceptions of mental illness has been demonstrated and appeals to the availability of *accepta ble* mental health care services. Mental healthcare providers’ sensitivity to cultural and religious beliefs will enrich the therapeutic relationship; hence healthcare providers should receive training focusing on the influence these belief systems have on patients’ perceptions of mental illness and their consequent help-seeking behaviour. Likewise, findings of the current study suggest a need for the incorporation of complementary and alternative treatment strategies in the rendering of mental healthcare services. Such inclusion acknowledges MHCCs’ preferences and may reduce the time required to obtain remission and recovery. Although its application has limitations, future research may provide useful insight for the formulation and implementation of interventions that MHCCs believe to be effective.

